# Compatibility of Maximum Inorganic and Organic Calcium and Phosphate Content in Neonatal Parenteral Solutions

**DOI:** 10.1038/s41598-019-46987-y

**Published:** 2019-07-19

**Authors:** Dorota Watrobska-Swietlikowska

**Affiliations:** Department of Pharmaceutical Technology, Medical University of Gdansk, 107 Hallera Ave., 80-416 Gdansk, Poland

**Keywords:** Paediatric research, Nutrition

## Abstract

The purpose of the study was to determine the maximum safe concentration of calcium and phosphate in neonatal parenteral nutrition (PN) solutions when various combinations of inorganic and organic salts are applied. Twelve PN solutions for neonatal use were aseptically prepared. Increasing concentrations of inorganic and organic calcium and phosphate were added to the standard formulas. Each admixture was separately tested according to the following conditions; after mixing, at 37 °C for 24 hr, and the maximum safe combination of calcium and phosphate were stored at 4 °C for 30 days and followed by 24 hr at 37 °C. Visual inspections and microscopic observation of undiluted PN solutions as well as the membrane filter after filtration of the PN solution, pH evaluation, and absorbance were examined. The safe maximum concentration of organic and inorganic calcium and phosphate were proposed individually for each composition of parenteral nutrition solutions. Surprisingly, organic calcium with organic phosphate showed precipitation but over the therapeutic range. The protective effect of amino acid was observed and higher concentrations of calcium and phosphate were free of precipitation. This work is valuable in daily practice as it allows an increase in the limits of calcium and phosphate in PN solutions for infants.

## Introduction

Neonatal and pediatric parenteral nutrition (PN) admixtures are very complex and difficult due to the small volume and numerous additives, making them highly concentrated solutions. The most important physical incompatibility in parenteral nutrition is the precipitation of calcium phosphate, particularly for preterm infants who require high concentrations of these electrolytes in a small volume^1^. The insoluble precipitate may result in serious clinical complications such as catheter occlusion or even microvascular pulmonary emboli^1^.

In parenteral nutrition, organic (calcium gluconate) and inorganic (calcium chloride) calcium salts are available. Chloride salt contains a higher proportion of calcium available to associate with free phosphate, leading to precipitation due to calcium chloride’s larger dissociation constant in comparison to calcium gluconate^2^. Furthermore, organic calcium salts have a lower degree of dissociation compared to inorganic calcium chloride^3^. These reasons cause that gluconate salt should be the predominant calcium source in compounding PN and it is only recommended in nutrition for preterm infants and children. In fact, there is a recommendation that an inorganic calcium salt such as calcium chloride should never be used in parenteral nutrition in children or in adults as well^4^. However, there are problems connected with using organic calcium salt. On the one hand, due to its better precipitation profile, organic calcium salt (calcium gluconate) is preferred in compounding parenteral admixtures, however, many authors suggest that it pollutes parenteral admixtures with aluminium^5,6^. Therefore, to minimize the aluminum content in PN admixtures, inorganic calcium salt is preferred.

Phosphate salts are inorganic (sodium phosphate) as well as organic (sodium glycerophosphate, NaGP) salts. Phosphate salts are supplied as a concentrated solution; NaGP contains 1 mmol/mL of phosphate whereas inorganic salts contain 2 mmol/mL of phosphate. NaGP is covalently bound to a glycerol backbone which hinders the formation of a precipitate with divalent calcium ions. The manufacturer reports the compound is stable in solutions compounded at concentrations up to 120 mmol/L NaGP (organic salt) and 96 mEq/L of calcium chloride. Dibasic calcium phosphate salt is 60 times less soluble than the monobasic form in an aqueous medium and its solubility is highly pH-dependant^7^. Furthermore, the use of organic phosphates considerably increases the calcium-phosphate compatibility. It was confirmed that the organic phosphate has superior stability in comparison to dibasic sodium phosphate regardless of pH, temperature, the type of calcium as well as amino acid or glucose concentrations^3,8^.

Many authors try to determine the maximum concentrations of calcium and phosphate at various combinations that can be mixed safely, but it cannot be universally determined due to the variety in the composition of compounding products^1–3,6,9^. Precipitation of calcium phosphate is much more frequent and dangerous in small patients (premature, newborn and children) because precipitation is more likely to occur at lower volumes and, moreover, is an endothermic reaction (incubators used for neonates increase the likelihood of precipitation). In fact, an increase in temperature will have two effects. One is the dissociation of calcium from its organic form; this will increase the availability of free calcium ions which will react with phosphate. Secondly, raising the temperature of a mixture may also shift the phosphate equilibrium from a mono- to a dibasic salt. However, some date confirms that NaGP and calcium chloride compatibility provides a clinical option for limiting aluminum contamination while providing sufficient calcium and phosphate to meet the needs of neonatal patients^9^.

Aluminum toxicity in parenteral nutrition solutions has been a problem for decades and is still unresolved. Europe lacks global legislation about an upper limit for aluminum contamination^10^. Parenteral nutrition solutions are contaminated with aluminum. Aluminum can cause osteomalacia in patients who receive long-term parenteral nutrition. It can also lead to encephalopathy in newborns and osteopenia in premature infants^4^. Borosilicate glasses, in particular, are known to have good chemical durability and are used commonly in the pharmaceutical industry as the primary container. However, these glass containers, even when made from borosilicate glass, can produce some undesirable effects that unavoidably occur from being in contact with drug solutions in long-term storage. Certain substances when stored in glass containers have a leaching action and aluminum can be released into the solutions and also aluminum can be present as a contaminant from some pharmaceutical products^11^. Parenteral nutrition products are contaminated with aluminum, especially those distributed in small-volume containers: calcium salts, trace elements, vitamins. The main source of aluminum contamination in parenteral nutrition is calcium gluconicum^12^. Solutions of trace elements and vitamins are usually ordered in small amounts, nevertheless, the high level of their contamination with aluminum makes them an important source^4^. The problem of aluminum contamination disappeared when plastic vials were used, however, plastic containers are more subjected to drug absorption. It is important to know that unaided visual observation of precipitation is limited to approximately 50 µm individual particles and can be highly variable. Subvisible precipitates ranging from 5 to 50 µm may occlude the microvasculature, such as in the pulmonary system. Particles below 5 µm, especially lipid globules, clog the pulmonary capillaries, producing an embolic syndrome and causing cell death mainly in pre-term infants^13^. Studies employing particle detection and size measurement by light obscuration provide objective evidence of subvisible microprecipitation which can be clinically dangerous^14^. Understanding the chemical and practical compatibility of calcium phosphate salts injections is critical to ensuring the safe intravenous administration of these supplements and preventing patient harm.

The purpose of the study was to assess the risk of calcium phosphate precipitation when various concentrations of inorganic and organic calcium and phosphate salts are mixed in various combinations in standard pediatric PN solutions. Our study assists practitioners in preventing calcium and phosphate precipitates in parenteral solutions.

## Results

### Visual inspection

**Figure 1:**
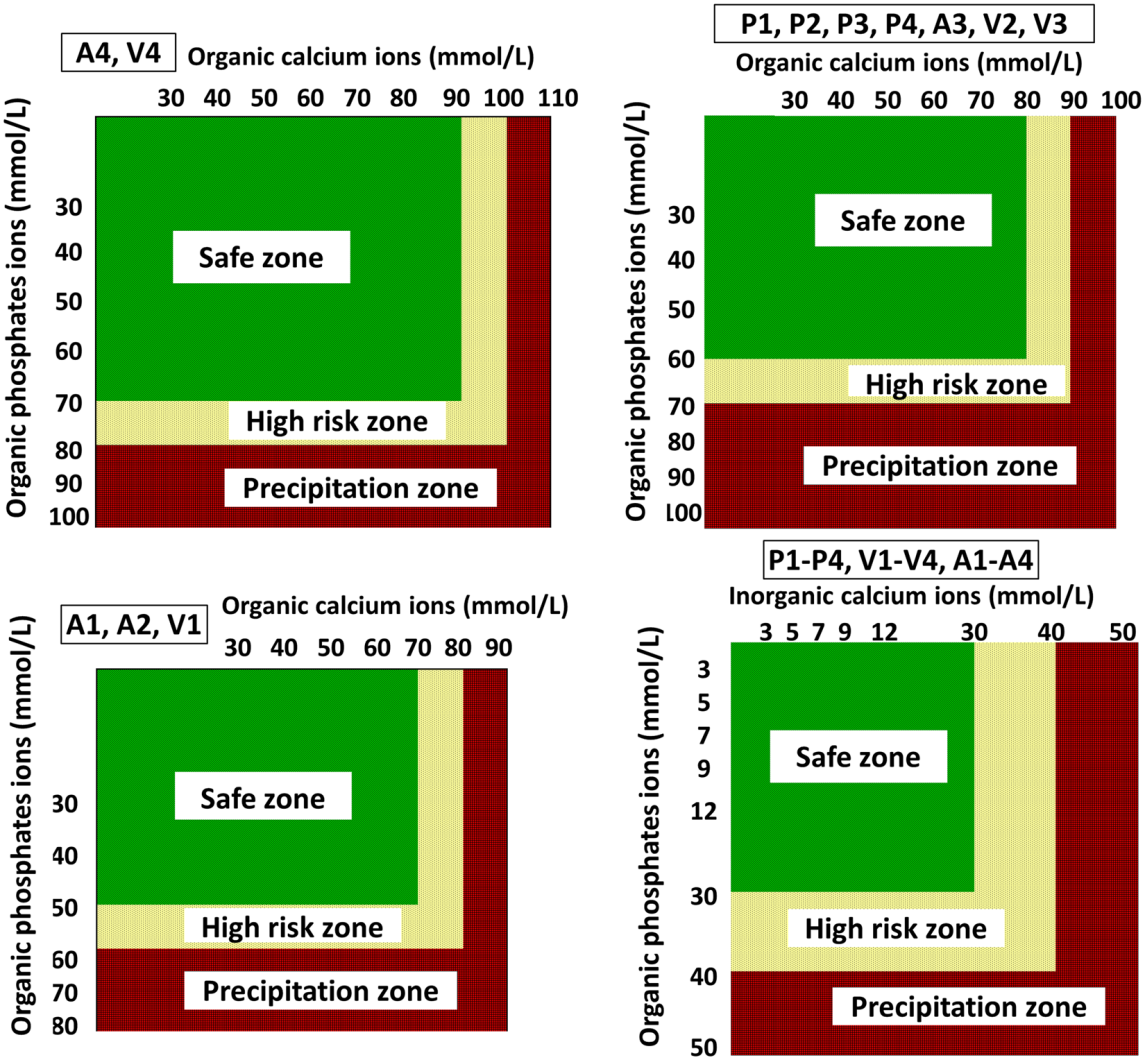
Stability and precipitation zones of PN solution in respect of the concentration of organic and inorganic calcium and organic phosphate ions (t = 24 hr, 37 °C).

In PN solutions P1, P2, P3, P4, A3, V2 and V3 the maximum concentration of organic ions of calcium and phosphate that did not cause opalescence after 24 hr at 37 °C was 80 mmol/L and 60 mmol/L, respectively. For PN solutions A4 and V4, the highest concentrations of organic salts were 90 mmol/L of calcium and 70 mmol/L of phosphate and for A1, A2 and V1 were 70 mmol/L and 50 mmol/L, respectively. Despite the fact that calcium and phosphate were organic salts, turbidity was observed, although it was above the therapeutic range (Fig. 1).

**Figure 2:**
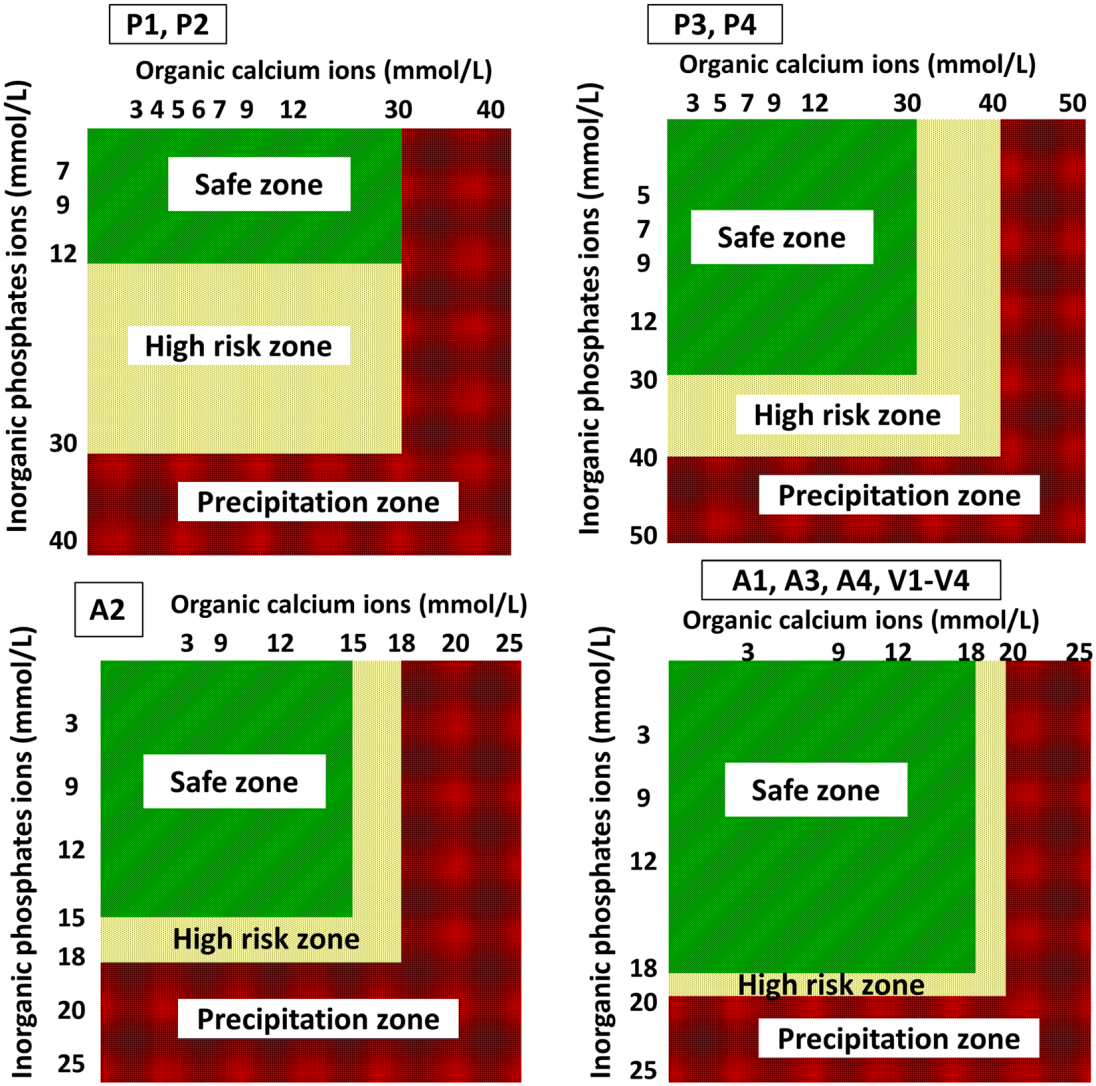
Stability and precipitation zones of PN solution in respect of the concentration of organic calcium and inorganic phosphate ions (t = 24 hr, 37 °C).

**Figure 3:**
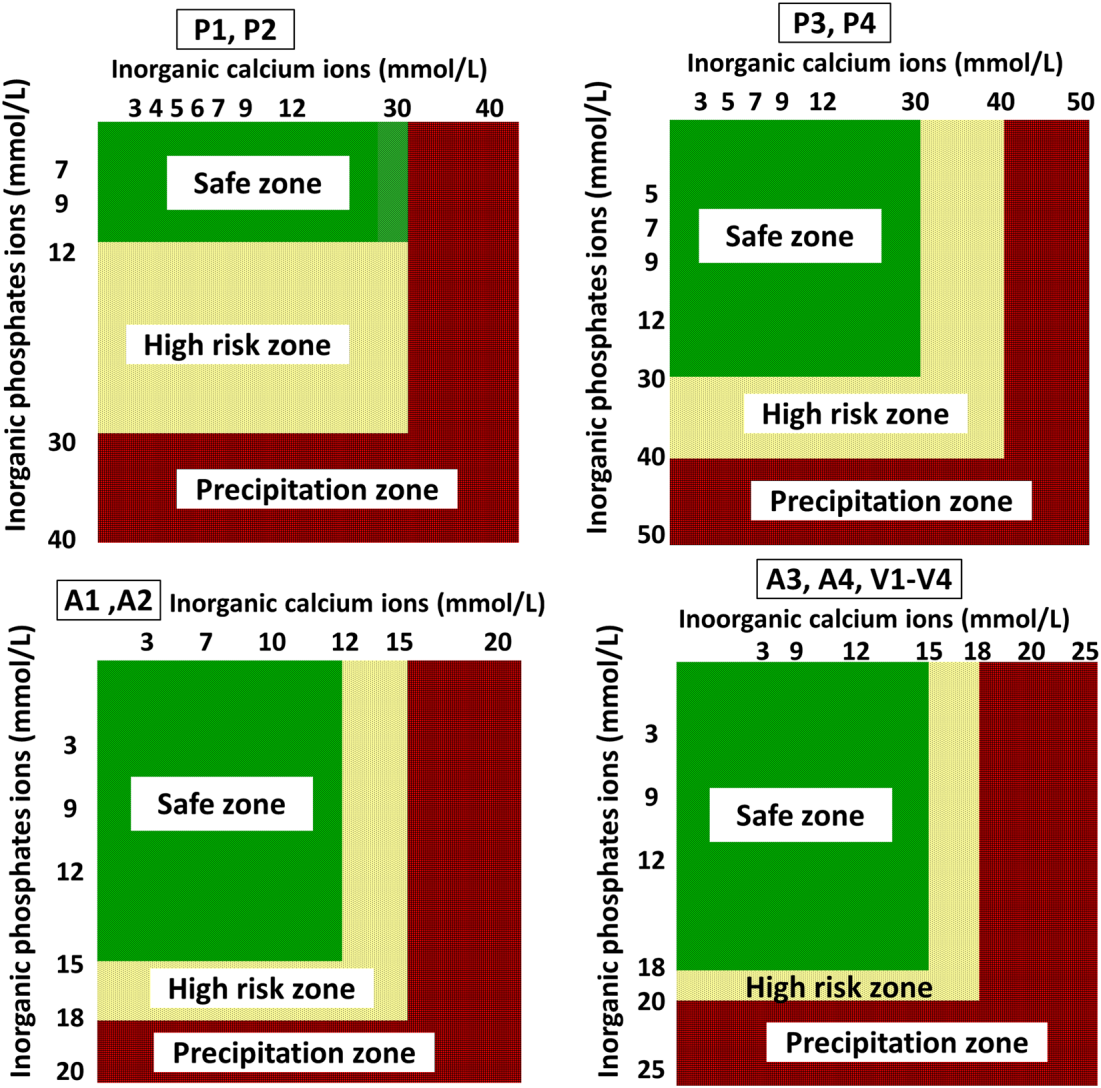
Stability and precipitation zones of PN solution in respect of the concentration of organic and inorganic calcium and organic phosphate ions (t = 24 hr, 37 °C).

A smaller concentration of organic calcium and inorganic phosphate showed precipitation (Fig. 2). In PN solutions P3 and P4, the maximum concentration of organic calcium and inorganic phosphate that did not cause opalescence after 24 hr at 37 °C was 30 mmol/L and 30 mmol/L, respectively, whereas in PN solutions P1 and P2 (a smaller content of glucose and amino acids) they were only 30 mmol/L and 12 mmol/L (Fig. 2). Using Aminoven or Vaminolact amino acids solutions allowed combining a much smaller calcium and phosphate content (Fig. 2). The same dependences were noticed when inorganic calcium and inorganic phosphate were used (Fig. 3).

After 30 days + 24 hr of storage, no changes were observed in the presence of organic calcium and organic phosphate ions in all the aqueous phases with the maximum safe (without precipitation after 24 hr) ion concentration, regardless of the amino acid preparation and composition (Tables 1–3). Other combinations of calcium and phosphate ions in the therapeutic range showed a characteristic white crystalline precipitate, falling to the bottom (Tables 1–3). This concerned PN solutions P1 and V1 in which inorganic calcium and phosphate ions were combined as well as P1, P2, P3, P4, A1, A3, A4 and V2 with organic calcium and inorganic phosphate ions. When mixing safe inorganic calcium salt with inorganic phosphate, nearly all PN solutions (except A1, V2 and V4) showed precipitation after 30 days + 24 hr of storage.

**Table 1:** Physicochemical Properties of PN Solution Composition P (n = 9; mean ± SD).

PN solution	Calcium [mmol/l]	Phosphate [mmol/l)	Time [days]	Visual observation	Absorbance	Microscopic observation	pH
P1	org 80	org 60	0	x	0.0019 ± 0.0001	x	7.18 ± 0.01
			31	x	0.0018 ± 0.0002	x	6.82 ± 0.00
	org 30	inorg 12	0	x	0.0034 ± 0.0001	x	5.82 ± 0.02
			31	ѵ	0.1134 ± 0.004	v	5.93 ± 0.01
	inorg 30	org 30	0	x	0.0037 ± 0.0001	x	6.37 ± 0.00
			31	x	0.0030 ± 0.0001	x	6.44 ± 0.01
	inorg 30	inorg 12	0	x	0.0052 ± 0.0002	x	5.86 ± 0.02
			31	v	0.1224 ± 0.008	v	5.41 ± 0.01
P2	org 80	org 60	0	x	0.0014 ± 0.0001	x	7.01 ± 0.02
			31	x	0.0014 ± 0.0002	x	7.01 ± 0.01
	org 30	inorg 12	0	x	0.0023 ± 0.0001	x	5.63 ± 0.01
			31	x	0.0017 ± 0.0001	x	5.82 ± 0.02
	inorg 30	org 30	0	x	0.0014 ± 0.0001	x	5.95 ± 0.00
			31	x	0.0017 ± 0.0002	x	6.29 ± 0.00
	inorg 30	inorg 12	0	x	0.0012 ± 0.0001	x	5.52 ± 0.01
			31	v	0.2131 ± 0.007	v	5.54 ± 0.01
P3	org 80	org 60	0	x	0.0014 ± 0.0001	x	6.84 ± 0.02
			31	x	0.0025 ± 0.0001	x	6.49 ± 0.02
	org 30	inorg 30	0	x	0.0081 ± 0.0003	x	5.53 ± 0.01
			31	v	0.1711 ± 0.009	v	5.58 ± 0.02
	inorg 30	org 30	0	x	0.0015 ± 0.0001	x	5.78 ± 0.01
			31	x	0.0031 ± 0.0001	x	6.00 ± 0.01
	inorg 30	inorg 30	0	x	0.0009 ± 0.0001	x	5.41 ± 0.00
			31	v	0.2155 ± 0.008	v	5.24 ± 0.01
P4	org 80	org 60	0	x	0.0009 ± 0.0001	x	6.79 ± 0.01
			31	x	0.0020 ± 0.0002	x	6.43 ± 0.01
	org 30	inorg 30	0	x	0.0005 ± 0.0001	x	5.68 ± 0.00
			31	v	0.2088 ± 0.008	v	5.51 ± 0.02
	inorg 30	org 30	0	x	0.0009 ± 0.0001	x	5.82 ± 0.01
			31	x	0.0013 ± 0.0001	x	5.94 ± 0.01
	inorg 30	inorg 30	0	x	0.0006 ± 0.0001	x	5.57 ± 0.01
			31	v	0.1899 ± 0.008	v	5.23 ± 0.01

x = no precipitation. v = precipitation.

**Table 2:** Physicochemical Properties of PN Solution Composition A (n = 9; mean ± SD).

PN solution	Calcium (mmol/l)	Phosphate (mmol/l)	Time (days)	Visual observation	Absorbance	Microscopic observation	pH
A1	org 70	org 50	0	x	0.0015 ± 0.0001	x	6.62 ± 0.02
			31	x	0.0079 ± 0.0002	x	6.49 ± 0.01
	org 18	inorg 18	0	x	0.0003 ± 0.0001	x	6.01 ± 0.00
			31	v	0.1773 ± 0.009	v	5.83 ± 0.01
	inorg 30	org 30	0	x	0.0052 ± 0.0001	x	6.44 ± 0.01
			31	x	0.0004 ± 0.0001	x	6.35 ± 0.01
	inorg 12	inorg 15	0	x	0.0010 ± 0.0002	x	5.90 ± 0.02
			31	x	0.0021 ± 0.0001	x	5.78 ± 0.01
A2	org 70	org 50	0	x	0.0080 ± 0.0001	x	6.80 ± 0.01
			31	x	0.0033 ± 0.0002	x	6.61 ± 0.01
	org 15	inorg 15	0	x	0.0009 ± 0.0001	x	6.11 ± 0.01
			31	x	0.0023 ± 0.0001	x	5.99 ± 0.02
	inorg 30	org 30	0	x	0.0008 ± 0.0002	x	6.42 ± 0.01
			31	x	0.0079 ± 0.0001	x	6.26 ± 0.01
	inorg 12	inorg 15	0	x	0.0019 ± 0.0001	x	6.06 ± 0.00
			31	v	0.1789 ± 0.007	v	5.90 ± 0.01
A3	org 80	org 60	0	x	0.0096 ± 0.0001	x	6.50 ± 0.01
			31	x	0.0062 ± 0.0001	x	6.39 ± 0.01
	org 18	inorg 18	0	x	0.0017 ± 0.0002	x	6.00 ± 0.01
			31	v	0.1109 ± 0.006	v	5.87 ± 0.01
	inorg 30	org 30	0	x	0.0037 ± 0.0001	x	6.25 ± 0.02
			31	x	0.0008 ± 0.0001	x	6.12 ± 0.02
	inorg 15	inorg 18	0	x	0.0004 ± 0.0001	x	5.91 ± 0.02
			31	v	0.1003 ± 0.008	v	5.79 ± 0.02
A4	org 90	org 70	0	x	0.0079 ± 0.0001	x	6.94 ± 0.01
			31	x	0.0060 ± 0.0001	x	6.70 ± 0.00
	org 18	inorg 18	0	x	0.0015 ± 0.0002	x	5.89 ± 0.01
			31	v	0.2091 ± 0.012	v	5.56 ± 0.01
	inorg 30	org 30	0	x	0.0059 ± 0.0001	x	6.20 ± 0.01
			31	x	0.0054 ± 0.0002	x	6.19 ± 0.02
	inorg 15	inorg 18	0	x	0.0005 ± 0.0001	x	5.85 ± 0.00
			31	x	0.0026 ± 0.0001	x	5.69 ± 0.01

x = no precipitation. v = precipitation.

**Table 3:** Physicochemical Properties of PN Solution Composition V (n = 9; mean ± SD).

PN solution	Calcium (mmol/l)	Phosphate (mmol/l)	Time (days)	Visual observation	Absorbance	Microscopic observation	pH
V1	org 70	org 50	0	x	0.0005 ± 0.0001	x	6.70 ± 0.01
			31	x	0.0088 ± 0.0002	x	6.55 ± 0.01
	org 18	inorg 18	0	x	0.0004 ± 0.0001	x	6.09 ± 0.01
			31	x	0.0006 ± 0.0001	x	5.83 ± 0.01
	inorg 30	org 30	0	x	0.0010 ± 0.0002	x	6.54 ± 0.01
			31	x	0.0013 ± 0.0001	x	6.31 ± 0.01
	inorg 15	inorg 18	0	x	0.0071 ± 0.0001	x	5.99 ± 0.01
			31	v	0.2132 ± 0.011	v	5.78 ± 0.01
V2	org 80	org 60	0	x	0.0062 ± 0.0001	x	6.57 ± 0.02
			31	x	0.0004 ± 0.0001	x	6.49 ± 0.01
	org 18	inorg 18	0	x	0.0006 ± 0.0002	x	5.95 ± 0.01
			31	v	0.1891 ± 0.009	v	5.80 ± 0.01
	inorg 30	org 30	0	x	0.0015 ± 0.0001	x	6.30 ± 0.01
			31	x	0.0029 ± 0.0001	x	6.05 ± 0.01
	inorg 15	inorg 18	0	x	0.0022 ± 0.0002	x	5.64 ± 0.00
			31	x	0.0099 ± 0.0003	x	5.56 ± 0.01
V3	org 80	org 60	0	x	0.0028 ± 0.0002	x	6.35 ± 0.01
			31	x	0.0031 ± 0.0001	x	6.21 ± 0.01
	org 18	inorg 18	0	x	0.0015 ± 0.0001	x	5.68 ± 0.01
			31	x	0.0007 ± 0.0001	x	5.60 ± 0.01
	inorg 30	org 30	0	x	0.0016 ± 0.0001	x	6.05 ± 0.02
			31	x	0.0055 ± 0.0002	x	5.90 ± 0.02
	inorg 15	inorg 18	0	x	0.0002 ± 0.0001	x	5.58 ± 0.02
			31	v	0.1193 ± 0.010	v	5.47 ± 0.02
V4	org 90	org 70	0	x	0.0072 ± 0.0003	x	6.80 ± 0.01
			31	x	0.0060 ± 0.0001	x	6.62 ± 0.01
	org 18	inorg 18	0	x	0.0003 ± 0.0001	x	5.79 ± 0.00
			31	x	0.0013 ± 0.0001	x	5.62 ± 0.01
	inorg 30	org 30	0	x	0.0082 ± 0.0002	x	6.19 ± 0.01
			31	x	0.0052 ± 0.0001	x	6.11 ± 0.01
	inorg 15	inorg 18	0	x	0.0022 ± 0.0001	x	5.64 ± 0.01
			31	x	0.0006 ± 0.0001	x	5.50 ± 0.02

x = no precipitation. v = precipitation.

Limitation: Analysis of samples with precipitation after 31 days of storage should be performed more frequently from t = 0 to t = 31 days to determine the point when precipitation occurred.

### Microscopic observation

**Figure 4:**
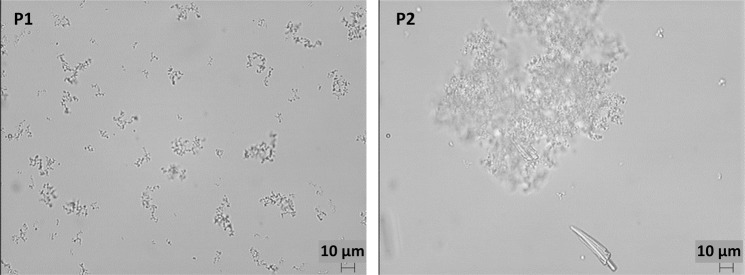
Amorphous precipitate of organic calcium (90 mmol/L) and organic phosphate (70 mmol/L) in PN solutions P1 and P2.

**Figure 5:**
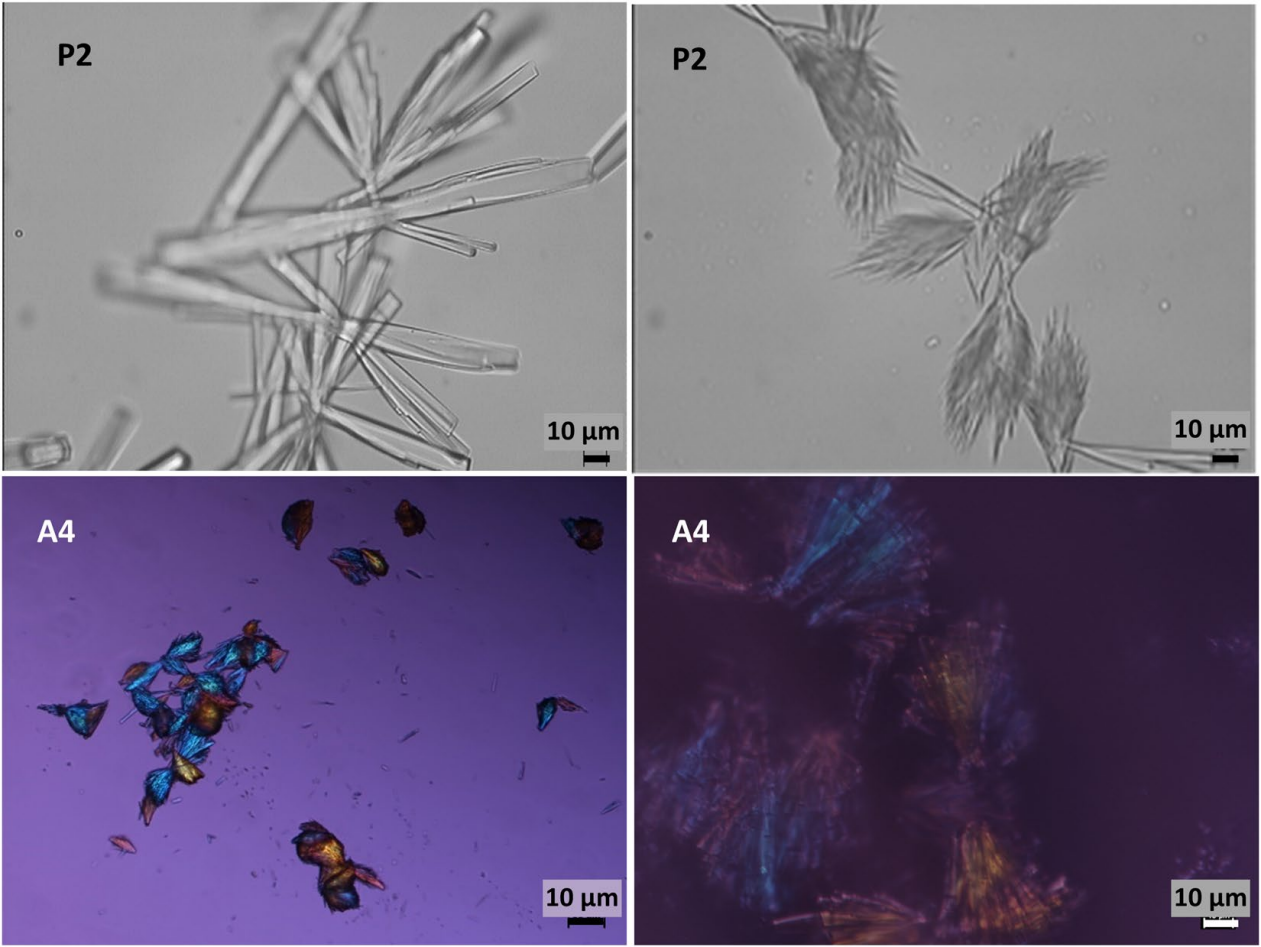
Crystal precipitation of inorganic calcium and inorganic phosphate in PN solutions P2 and A4.

Microscopic observations of the PN solutions confirmed the visual observations (Tables 1–3). The amorphous, spongy precipitate, forming larger agglomerates was present in PN solutions with organic calcium and phosphate ions at concentrations above the therapeutic range (Fig. 4). Crystal precipitation in the range of 50 to 100 μm in size was visible in PN solutions containing inorganic ions (Tables 1–3). The crystals appeared individually in the field of view or formed compact structures (Fig. 5). Microscopic observations of precipitation in PN solutions were compared with placebo solutions which indicate that precipitation was formed of calcium and phosphate ions.

### pH measurement

The pH of the PN solutions ranged from 5.23 to 7.18 (Table 1). Higher concentrations of organic calcium and phosphate ions were characterized by the highest pH values. There was no relationship between the presence of precipitation and a change in pH values. A statistically significant decrease (p < 0.05) from the baseline (t = 0) of each composition of the PN solution to 30 days + 24 hr was observed (Tables 1–3), which possibly resulted from the removal of HPO4^2−^ (monohydrogen phosphate) by the reaction with calcium or the unmeasured degradation of amino acids and glucose.

### Absorbance measurement

PN solutions with calcium and phosphate with no visual changes as well as with turbidity were examined in the visible light range. Absorbance at 600 nm was in the range of 0.0001–0.0120 (Tables 1–3) for most of the samples. Absorbance above 0.1000 was measured for turbidity samples with precipitate (Tables 1–3).

### Risk curves

Using spectrophotometric measurement as well as visual and microscopic observation, maximum, safe for therapeutic use, calcium and phosphate ions concentrations were determined to ensure no precipitation at t = 24 hr (37 °C). Figures 2–4 show the risk curves of precipitation in the tested PN solutions, depending on the type and the concentration of calcium and phosphate ions. The safe zone (green) includes the range of ion concentrations at which precipitation did not occur within 24 hours at 37 °C after combining. The high risk zone (orange) means the concentration range that was not measurable. There were different limit concentrations at which no precipitation occurred after 24 hours. Limit concentrations depended on the composition of the aqueous phase. Further increases in ion concentration caused precipitation (red zone).

## Discussion

Maximizing calcium and phosphate ion concentrations without increasing the risk of precipitation is a challenge for manufacturers of PN solutions, particularly in neonatology. Indeed, premature neonates have very high calcium (Ca) and phosphate (PO4) requirements for bone mineralization, often have a restricted fluid intake and many parenteral solutions are compounded close to saturation point^15^. This limits the practitioner’s ability to adequately supply the required amounts of calcium and phosphate, especially in small patients. Furthermore, varying the concentration of other components of the parenteral solution such as amino acids, glucose, electrolytes and lipids can alter the saturation point, potentially increasing the probability of precipitation.

The nature of calcium and phosphate salts directly influences the solubility of calcium phosphate. Given the differences in dissociation characteristics, the relative concentrations of calcium or phosphate available for precipitation are higher when inorganic salts are used^1^. The general suggestion when compounding parenteral solutions, especially for premature infants, is to use organic phosphate (glycerophosphate) together with organic calcium salts (calcium gluconate) as an uncomplicated and safe way to simultaneously administer high amounts of calcium and phosphate in total PN solutions^3^. Organic calcium salts such as calcium gluconate (Ca-glu) are widely used but are temporarily unavailable due to drug shortages. The availability of organic phosphate salts (sodium glucose-1-phosphate, G1P as well as sodium glycerophosphate, NaGP) is also limited because they are not registered and approved for use in every country^16,17^. In hospital practice, it is very valuable to know if electrolyte replacement will be safe for patients. Due to the various combinations in the composition of parenteral admixture solution for small patients and a variety of calcium and phosphate salts available, it is very difficult to establish safe limits of calcium and phosphate concentrations.

In this study, the experimental information provided is useful for optimizing the hospital compounding of PN solutions containing calcium and phosphate ions. The solubility of calcium phosphate depends on several variables such as the pH or the concentration of amino acids and glucose, however, the most relevant factor is the nature of the salt (organic or inorganic). It is impossible to establish rules to predict precipitation and each composition should be studied individually. Our study was performed in various conditions initially from those particularly prone to precipitation (low concentration of amino acids and glucose – composition “1”). Our data provide evidence that even in these worst-case conditions, a wide range (above the therapeutic range) of PN solutions with up to 80 mmol/L of organic calcium ions and 60 mmol/L of organic phosphate ions are stable for 30 days at 4 °C and following 24 hr at 37 °C. Consequently, the limits included in our prescribing software were increased, allowing the prescription of compositions of PN solutions with calcium and phosphate ions up to 80 mmol/L and 60 mmol/L, respectively. This study helps physicians in daily practice who are no longer limited in their prescriptions of calcium and phosphate ions. A second useful aspect of this study is the economic advantage – the designated safe zones in which precipitation does not occur allows the use of inorganic salts which are much cheaper than organic calcium and phosphate salts. The third advantage is that it overcomes the shortage of organic salts which very often occurs in hospital practice and prevents the safe administration of PN solutions.

Most investigators identify calcium phosphate precipitation by using visual inspection. However, a visual inspection can sometimes be ineffective in preventing precipitate in parenteral solution, especially for all-in-one admixtures in which lipid emulsions are present or when calcium phosphate precipitation is not enough to be visible (at the beginning of this process). The second limitation is the fact that visual inspection is very subjective and does not allow distinguishing calcium phosphate precipitation from other precipitation. Other analytical methods should be applied to ensure safe therapy for patients.

Light microscopy can be used to detect the particle counts in solutions as well as on filter surfaces after filtration of the PN solution^7^. The isolation of particles by filtration of the PN solution followed by microscopic observation of the filter surfaces is time-consuming but is a relatively sensitive method. It should be kept in mind that it depends on particle suspension before filtration and it also does not differentiate between calcium phosphate precipitation and other particles present in the mixture. To minimize that problem, placebo solutions were prepared with calcium and phosphate ions without other components to provoke precipitation and the shape and size of precipitation were compared.

The absorbance from the UV-Vis spectrophotometer to determine the presence of precipitation was also involved^8^. An absorbance greater than 0.015 at 600 nm was considered evidence of precipitation^18^.

The maximum concentrations of calcium and phosphate ions without precipitation up to 24 hours at 37 °C were established for each tested composition of PN solution. Our results guarantee safe parenteral therapy for neonates. The focus was on salt combinations containing at least one type of ion in an inorganic form. In addition, the minimum concentrations of organic calcium and phosphate salts which cause precipitation were studied. For this case, the salts were tested in ranges much higher than the therapeutic values. The composition of PN solutions were the ones most commonly used in clinical practice. Three different pediatric amino acids solutions (Primene, Aminoven and Vaminolact) were used. The PN solutions varied in their glucose and amino acid concentrations. In addition, the studied solutions were stored for up to 30 days to check the effect of time on their properties. The results confirmed the protective effect of amino acids on PN solutions – with a higher concentration of amino acids, no precipitation was observed despite the high concentrations of calcium and phosphate ions (Tables 1–3). In PN solutions with a lower concentration of amino acids, the presence of a precipitate in the same ion range was noted (P1 and P2 vs. P3 and P4).

Inorganic calcium and phosphate salts were characterized by a higher risk of precipitation than organic salts (Figs 1–3). For a combination of inorganic calcium salts and organic phosphate salts, it was possible to use higher concentrations than in the reverse case (Figs 1–3). The generally accepted principle regarding organic calcium and phosphate ions is that they can be combined without restrictions because no precipitation will occur. It was shown that there is a risk of precipitation, nevertheless, it occurs outside the therapeutic range of concentrations (calcium ions 50 mmol/L, phosphate ions 30 mmol/L) (Figs 1–3). The pH values increased with the increasing concentration of organic ions (Tables 1–3). However, the studies showed a significant decrease in the pH value with the storage time for aqueous phases with organic calcium and phosphate ions (Tables 1–3). However, the presence of precipitation has no influence on this parameter.

Only a combination of various technical and time-consuming methods used by laboratories could ensure detection of precipitation in a PN solution. However, in hospital practice, using a relatively simple method such as visual examination, with appropriate illumination and background, should always be applied as it as reliable as a general screening method.

## Conclusion

The maximum safe combination of calcium and phosphate for each investigated composition of PN solution, even in the worst-case situations, was proposed. This work is valuable in daily practice as it allows an increase in the limits of calcium and phosphate in PN solutions for infants. This study provides valuable data on the compatibility of inorganic and organic calcium salts with inorganic and organic phosphate salts in various combinations in PN solutions compounded for neonates. This data adds to the literature information which may be used to evaluate different options for administering calcium and phosphate in neonatal PN solutions. This may be valuable information when there is a shortage of PN organic additives of calcium and phosphate. It should be pointed out that any change in the composition of PN solutions needs a new analysis.

## Methods

### Composition and preparation of PN solutions

Twelve basic (without calcium and phosphate salts) PN solutions were prepared aseptically following international recommendations under a laminar airflow hood in a class A horizontal laminar-airflow hood, located in a GMP class B clean room at the Stanley Dudrick’s Memorial Hospital in Skawina, Poland. Single-chamber, monolayer ethylene vinyl acetate bags, Exacta-Mix Eva Bag Parenteral, constituting the packaging of PN solutions was used. For this purpose, a Baxa 24 computer-controlled mixer was applied, allowing for precise transfusion following base fluids, i.e., 40% dextrose solution (B. Braun Melsungen, Germany), amino acids solution (Aminoven Infant 10% or Vaminolact, Fresenius Kabi, Uppsala, Sweden or Primene 10%, Baxter Dutschland GMBH, Germany), water for injections, sodium chloride (Natrium chloratum 10%, Polpharma, Starogard Gdański, Poland), potassium chloride solution (Kalium chloratum 15%, WZF Polfa, Warsaw, Poland), magnesium sulfate solution (Inj. Magnesii sulfurici 20%, Polpharma, Starogard Gdański, Poland) and trace elements (Peditrace, Fresenius Kabi, Uppsala, Sweden). No lipids were added because they would obscure the presence of a precipitate. All products in this study were approved for use in Poland for parenteral nutrition. Each of the twelve of PN solutions was prepared triplicate and was tested for compatibility using various concentrations of amino acid solutions and glucose (Table 4).

**Table 4:** Composition of PN Solutions under Test.

PN solution	Amino acids solution	Amino acid (g/L)	Glucose (g/L)	Na^+^ (mmol/L)	K^+^ (mmol/L)	Mg^2+^ (mmol/L)	Peditrace (ml/L)	Total volume (ml)
P1	Primene 10%	10	50	200	100	10	15	500
P2	Primene 10%	10	200	200	100	10	15	500
P3	Primene 10%	40	50	200	100	10	15	500
P4	Primene 10%	40	200	200	100	10	15	500
A1	Aminoven 10%	10	200	200	100	10	15	500
A2	Aminoven 10%	10	50	200	100	10	15	500
A3	Aminoven 10%	40	50	200	100	10	15	500
A4	Aminoven 10%	40	200	200	100	10	15	500
V1	Vaminolact	10	50	200	100	10	15	500
V2	Vaminolact	10	200	200	100	10	15	500
V3	Vaminolact	40	50	200	100	10	15	500
V4	Vaminolact	40	100	180	100	10	15	500

### Storage and sampling

To analyze the compatibility of calcium and phosphate, in the first step 100 mL of each basic PN solution was transferred to glass bottles, increasing organic or inorganic calcium (Calcium gluconate solution 10%, Monico SPA, Mestre, Venezia and Calcii chloratum 10%, Polfa Warszawa, Warsaw, Poland, respectively) and phosphate (Glycophos, Fresenius Kabi, Uppsala, Sweden and Addiphos, Fresenius Kabi, Uppsala, Sweden, respectively) concentration above therapeutic use were added.

Each of the 12 PN solutions with various combinations of calcium and phosphate was tested in triplicate. All samples with an increasing concentration of calcium and phosphate ions (the procedure of preparing the samples with increasing concentration of these ions is described in *Data interpretation*) were stored in glass bottles for 24 hours at 37 °C prior to analysis. Solutions were placed in a thermostatic chamber at 37 °C (without any light) for 24 hours to simulate pediatric conditions similar to body temperature. In the second step, the samples with the maximum safe (meaning that is mean without any precipitation after 24 hr) concentration of calcium were stored in monolayer ethylene vinyl acetate bags (the same used for preparing PN solutions) for 30 days at 4 °C followed by 24 hr at 37 °C under regular light conditions, without any light protection. At the beginning (t = 0) and at the end (t = 31) of the storage period, physical analyses were performed.

### Evaluation of physicochemical stability

The physical analyses were performed at t = 0 and 24 hr (samples with increasing calcium and phosphate concentration) and at t = 0 and 30 days + 24 hr (samples with the maximum safe calcium and phosphate concentration determined in the first step of the study, after 24 hr). The study was performed with three different aliquots of each PN solution under test. To guarantee the homogenization of solutions after storage for 24 hr as well as 30 days + 24 hr, the samples were gently agitated before the analysis.

#### Visual observation

The samples were visually inspected against a black and white contrast background for evidence of precipitation.

#### Microscopic observation

Each PN solution with an increasing concentration of calcium and phosphate was checked microscopically by a 100× light optic microscope with a camera (B1 223 A Motic, Wetzlar, Germany) for microcrystals. Using the recommendations of European Pharmacopoeia (Ph Eur) for particle count, solutions were considered physically compatible if the crystal count was less than 12 particles per mL measuring ≥10 µm and no more than 2 particles per mL measuring ≥25 µm in diameter. 100 mL of each of the PN solutions with the maximum safe concentration of calcium and phosphate salts in different combinations were infused through a 0.45-micron nitrocellulose filter disk (Millipore, Billerica, MA) were evaluated for crystal content. Each disk was analyzed quantitatively for crystal precipitates under 100× magnification using a linear scale. Physically compatible solutions could contain no more than 2400 particles measuring ≥10 µm in diameter and 400 particles measuring ≥25 µm in diameter.

#### Quality analyzing of calcium phosphate crystals

Crystals observed after filtration of PN solutions were compared (taking into account the shape and size of precipitation), using microscopic observation, with crystals obtained in placebo solutions which contained only calcium and phosphate salts in different combinations to provoke precipitation.

#### pH measurement

The pH values of the PN solutions were determined at 25 °C (pH-Meter type 350, Orion-Research, Boston, USA) with a combined electrode (Type ERH-11, Hydromet, Warsaw, Poland). Before each pH measurement, a two-point calibration of the pH meter was performed, each with buffer solutions of pH 7.00 and pH 4.00, respectively. The pH 7.00 solution was used afterwards as a control. Between the calibration steps, the electrode was rinsed with distilled water and wiped dry. Each sample was triplicate measured after 3 min of equilibration.

#### Light scattering

PN solutions were examined for light scattering by a UV-Vis spectrophotometer JASCO V-530 (Jasco International CO. LTD., Tokyo, Japan) at 600 nm against sterile water for injection blank. An absorbance of greater than 0.015 was determined as the “arbitrary threshold for precipitation”.

### Data interpretation

The results were classified in three categories: “stability zone” for clear samples without precipitation, “high risk zone” for areas of concentration without measurements and “precipitation zone” when visible particles or precipitates were detected. Increasing step by step quantities of calcium and phosphate in the four different combinations (inorganic and organic salts) in concentrations ranging from 1 to 110 mmol/L depending on the composition of the basic PN solution were used to establish calcium phosphate precipitation zones.

When inorganic salts were used, concentrations were increased in steps of 1 mmol/L for each salt (Ca and PO_4_), beginning at 1 mmol/L and continuing to 30 mmol/L, then 10 mmol/L were added to refine the curve to 50 mmol/L. When organic salts were used, the steps between the two concentrations were 10 mmol/L of each salt, beginning at 10 mmol/L and continuing, depending on the composition, to 50 or even 110 mmol/L.

### Statistical analysis

The results are presented as mean and standard deviation (SD). The results were evaluated using the non-parametric ANOVA Friedman test. Statistica 13 software (StatSoft, Kraków, Poland) was used. The priori level of significance was 0.05.

## Data Availability

At the present stage I have no supplementary information accompanying this manuscript. However, I am happy with publishing such information if necessary.
